# Trauma-informed training as a path to intergroup readiness: a mixed-methods evaluation of EMDR education in a shared society context

**DOI:** 10.3389/fpsyg.2025.1618634

**Published:** 2025-11-20

**Authors:** Dorit Segal

**Affiliations:** EMDR for Peace, Pardes-Hanna, Israel

**Keywords:** EMDR therapy, trauma-informed training, intergroup dialogue, reconciliation, peacebuilding, shared society, therapist education, conflict transformation

## Abstract

Trauma-informed therapies, particularly Eye Movement Desensitization and Reprocessing (EMDR), have demonstrated individual efficacy, yet their broader societal impacts remain underexplored. This mixed-methods study examined how participation in a shared EMDR training program influences professional self-efficacy, intergroup openness, and reconciliation readiness among therapists working within a conflict-affected society. Twenty-four participants completed a post-training questionnaire that included quantitative Likert-scale items and qualitative reflective prompts. Descriptive and exploratory analyses indicated overall positive shifts in perceived professional growth, hope for reconciliation, and cross-group helping capacity, alongside greater variability in reported interpersonal understanding. Exploratory subgroup analyses compared Jewish-Israeli and Arab-Palestinian participants, revealing meaningful but non-significant trends reflecting each group’s distinct sociocultural perspectives. Thematic analysis of qualitative responses identified four main themes: interpersonal connection, experiential learning, hope mixed with skepticism regarding peacebuilding, and suggestions for enhancing intergroup dialogue opportunities. Together, these findings highlight that trauma-informed professional training can simultaneously support clinical development and foster initial steps toward intergroup empathy and social healing. Intentional facilitation of cross-group engagement remains critical to maximize the broader peacebuilding potential of such initiatives, and future research should examine longitudinal and comparative outcomes across diverse program settings.

## Introduction

Trauma-informed therapies, and EMDR in particular, have demonstrated significant effectiveness in individual trauma recovery. However, their role in shaping intergroup dynamics and professional identity within divided societies remains underexplored.

In regions of protracted conflict, such as Israel-Palestine, therapists often work not only with personal trauma but also with the embedded sociopolitical contexts that surround it. While the therapeutic process is typically considered intrapersonal, the training of therapists itself can become a site of intergroup encounter—particularly when trainees come from communities with historical tensions.

EMDR for Peace, a nonprofit initiative, has been developing training programs that bring together Jewish and Arab participants—including those from Israel, the West Bank, East Jerusalem, and Gaza. These trainings aim to equip therapists with clinical skills while fostering shared spaces of learning, regulation, and professional solidarity.

In addition to intrapersonal trauma processing, collective identity and group narratives have been shown to play a central role in shaping individuals’ openness to reconciliation efforts. Research in peace psychology and conflict resolution suggests that the salience of group identity can influence both the psychological accessibility of hope and the perceived feasibility of intergroup healing (Bar-Tal, 2000; Volkan, 2006; Hammack, 2006). Thus, examining potential subgroup variations within shared therapeutic training contexts may yield important insights into the interplay between individual trauma recovery and collective sociopolitical dynamics.

This study explores how participation in EMDR training programs—particularly those conducted in binational and bilingual settings—may influence participants’ professional confidence, intergroup openness, and perceptions of peacebuilding potential through trauma work. In addition to professional development, such joint training contexts may offer a lens through which broader processes of intergroup reconciliation can be examined.

Emerging research in peace psychology suggests that individuals’ experiences of trauma healing and openness to reconciliation may vary across identity groups, particularly in contexts of protracted conflict (Bar-Tal, 2000; Volkan, 2006). Exploring potential subgroup differences in shared professional training settings offers an opportunity to deepen understanding of how trauma-informed practices can support not only individual resilience but also collective reconciliation processes.

## Research question

How does participation in EMDR-based training, provided through a shared society framework, affect participants’ attitudes toward intergroup cooperation and their perceived role in social healing?

This article builds on previous EMDR for Peace initiatives, which have shown the potential of EMDR-based interventions to foster intergroup empathy and psychological safety. In contrast to one-time workshops, this study explores sustained engagement through structured therapist training. The aim is to assess how such professional programs may influence not only clinical competence but also attitudes toward peacebuilding and shared identity.

Grounded in the same theoretical foundations—Adaptive Information Processing (Shapiro, 2001), trauma-informed group facilitation (Jarero et al., 2013; Maxfield, 2021), and peace psychology (Bar-Tal, 2000; Lederach, 1998)—this work continues to explore how trauma treatment and social healing may intersect within divided societies.

## Materials and methods

### Participants

A total of 24 mental health professionals and trainees participated in the study. All had completed EMDR training organized by EMDR for Peace during 2024–2025 within a binational, bilingual framework. Participants were drawn from diverse professional and cultural backgrounds across Israel and Palestine.

Demographically, 12 participants (50%) identified as Jewish-Israeli and 12 (50%) as Arab-Palestinian, including Muslim (*n* = 8), Christian (*n* = 1), and individuals describing themselves as following other or independent spiritual practices (*n* = 3). Gender and age data were collected but are not reported here to preserve anonymity, as participants were drawn from small, interlinked professional communities.

All participants held at least a Master’s degree in a therapeutic field (e.g., psychology, social work, art therapy, or psychiatry) and met the professional eligibility criteria for EMDR training as set by EMDR Europe. They had completed the basic EMDR training (Level 1 and/or Level 2) through EMDR for Peace and were either licensed clinicians or advanced trainees in psychotherapy-related disciplines.

Participation was voluntary and uncompensated. Respondents were invited to complete the anonymous post-training questionnaire at the end of the training. No identifying information was collected, ensuring full confidentiality.

Due to the anonymous design, no detailed sociodemographic or geographic identifiers were collected; future studies will include more comprehensive demographic data.

Table 1 presents the demographic composition of the sample. This balanced and heterogeneous sample reflects EMDR for Peace’s mission to foster joint professional development among Jewish-Israeli and Arab-Palestinian therapists within a shared-society framework.

**TABLE 1:** Participant demographics (*N* = 24).

Variable	Categories	*N*	%
Cultural/religious identification	Jewish-Israeli	12	50
	Arab-Palestinian (Muslim = 8; Christian = 1; Other spiritual = 3)	12	50
Professional background	Social worker varied	Varied	–
	Psychologist	Varied	–
	Psychiatrist	Varied	–
	Art therapist	Varied	–
EMDR training level	Level 1 + 2 + certification prep	Majority (∼65%)	–
	Level 1 or 2 only	Remaining (∼35%)	–

Exact professional and training-level numbers are omitted to preserve anonymity. Due to the anonymous design, detailed sociodemographic and geographic data were not collected. Percentages are rounded to whole numbers.

### Procedure

Participants were recruited from two binational EMDR training courses organized by EMDR for Peace during 2024–2025, conducted within bilingual learning environments. The training followed the official EMDR Europe curriculum and included both Level 1 and Level 2 components.

Each level consisted of three full training days, comprising 4 h of theoretical instruction and 4 h of supervised practicum per day. Following the initial training, participants attended four group supervision sessions (3 h each), focused on the application of EMDR principles to their clinical work. The Level 2 structure mirrored that of Level 1.

One training cohort was conducted fully online via Zoom, while the second took place in person, with all supervision sessions delivered online. Instruction was primarily in English, with translation and linguistic support available in Hebrew and Arabic as needed. The official course manual was provided in English, and supplementary translated materials were made available in Hebrew and partially in Arabic.

The training combined didactic lectures, pre-recorded video demonstrations, and live demonstrations conducted by the trainer with volunteer participants. Followed by supervised practicum sessions, in which participants practiced EMDR techniques with one another under the supervision of certified EMDR facilitators. Supervision sessions provided space for reflection, case discussions, and integration of learning into participants’ professional contexts.

At the completion of all training and supervision phases, participants were invited to complete an anonymous post-course questionnaire designed as part of EMDR for Peace’s internal evaluation process. The invitation was distributed via the course WhatsApp group with an open link to the survey. Participation was voluntary and uncompensated, and completion of the questionnaire implied informed consent.

A total of 24 out of 31 participants responded to the questionnaire, representing a response rate of approximately 77%. No identifying information was collected, ensuring full confidentiality.

### Measures

The questionnaire was developed specifically for this study as part of EMDR for Peace’s internal evaluation process. It was designed collaboratively by two EMDR trainers and two peacebuilding practitioners who are actively involved in the organization’s operations and long-standing fieldwork in binational dialogue and trauma recovery. Their combined experience in training, organizational assessment, and cross-community facilitation guided the content and structure of the tool.

The questionnaire was written in English, the working language of the training, and included both quantitative and qualitative components.

The quantitative section consisted of four Likert-scale items rated from 1 (Strongly Disagree) to 7 (Strongly Agree), assessing participants’ perceptions in the following domains:

- Belief in trauma healing as a path to reconciliation.
- Deepened understanding of people from different backgrounds.
- Increased perceived capacity to help across group lines.
- Hope for change through shared therapeutic work.

Participants also responded to three open-ended reflective questions, inviting them to elaborate on their learning and intergroup experience:

- What was the most meaningful part of your training experience?
- Did participating in the EMDR training affect the way you think about peacebuilding or intergroup work? If so, how?
- Is there anything you would like to see added or changed in future joint trainings?

The combination of scaled and narrative questions was intended to capture both measurable trends—such as perceived professional readiness and intergroup openness—and the subjective meanings participants attributed to their shared training experience.

This mixed-methods design enabled a richer and more nuanced understanding of the complex and sensitive processes emerging in joint trauma-focused training, beyond what could be reflected through quantitative data alone.

The full English version of the questionnaire is provided in Appendix A.

### Data analysis

Given the sensitive and multifaceted nature of intergroup encounters in conflict-affected settings, a mixed-methods approach was adopted to capture both measurable and experiential aspects of participants’ learning. Quantitative and qualitative analyses were conducted separately and then interpreted in relation to one another to provide a comprehensive understanding of the data.

### Quantitative analysis

Quantitative data were analyzed using descriptive statistics (means, standard deviations, and response distributions) to explore patterns in professional self-efficacy, intergroup openness, and perceived readiness for collaborative work.

All analyses were performed using SPSS (Version 29). No inferential or significance testing was conducted due to the modest sample size (*N* = 24).

Exploratory group comparisons were visualized to illustrate trends in responses between Jewish-Israeli and Arab-Palestinian participants, with particular attention to hope-related items.

### Qualitative analysis

The qualitative data—participants’ open-ended narrative reflections—were analyzed using thematic analysis following the six-phase framework outlined by Braun and Clarke (2006):

(1) data familiarization, (2) initial coding, (3) theme generation, (4) theme review, (5) theme definition and naming, and (6) final reporting.

The first author, who has formal training in qualitative research and EMDR supervision, conducted the initial coding.

To enhance credibility, a second reviewer independently coded a random subset of 20% of the responses. Discrepancies were discussed until consensus was reached, and themes were refined through iterative dialogue.

Thematic saturation was determined when no new themes emerged in successive rounds of review.

For each major theme, the analysis identified representative quotes and noted the proportion of participants whose responses reflected that theme, to ensure transparency and traceability.

### Integration of findings and transition to results

Findings from both strands of analysis were integrated during interpretation to illuminate how measurable changes and personal reflections complemented one another.

The quantitative results provided an overview of trends in participants’ attitudes and perceived competencies, while the qualitative analysis added depth by illustrating the personal and relational meanings underlying those shifts.

The following section presents the results of both analyses, beginning with descriptive quantitative findings, followed by the qualitative themes that further contextualize and enrich the understanding of participants’ experiences.

### Ethical considerations

Participants provided informed consent prior to completing the questionnaire.

Participation was entirely voluntary and uncompensated, and had no impact on participants’ eligibility for training, completion status, or professional evaluation.

Data collection was fully anonymous and confidential, with no identifying information recorded and no individual clinical assessment conducted.

Ethical approval was not required according to Israeli institutional and national guidelines for non-clinical, anonymous educational research.

Nevertheless, the study was conducted in accordance with the ethical principles of the American Psychological Association (American Psychological Association [APA], 2017) and the Declaration of Helsinki (2013 revision) (World Medical Association, 2013), ensuring respect for participants, confidentiality, and responsible data management throughout the research process.

### Theoretical framework

This integrative framework—combining trauma-processing theory, peace psychology, and intergroup contact—guided both the design and interpretation of the study.

The present study is guided by an integrative theoretical framework that brings together trauma-processing theory, peace psychology, and intergroup contact theory to explain how professional EMDR training conducted in divided societies may influence both individual and relational dimensions of healing.

This study is grounded in the Adaptive Information Processing (AIP) model (Shapiro, 2001), which serves as the foundational theory of EMDR therapy. According to AIP, unprocessed traumatic experiences are stored in a dysfunctional state, retaining the original emotions, sensations, and cognitions associated with the event, and may be reactivated by subsequent triggers. EMDR therapy seeks to facilitate the reprocessing of these memories, allowing for the integration of adaptive information and the emergence of more functional emotional and cognitive responses. In recent years, EMDR has expanded beyond one-on-one clinical settings to include group protocols (Jarero et al., 2013; Maxfield, 2021) and community-based interventions in crisis and post-conflict contexts (Hasandedić-Đapo, 2018; Robinson and Kaptan, 2023).

While AIP theory focuses on the integration of fragmented traumatic memories at the individual level, its mechanisms—particularly adaptive reprocessing and emotional regulation—may extend to collective settings. When such processes occur in binational group contexts, they can foster shared regulation, empathy, and mutual recognition, thus linking individual healing to intergroup transformation.

The concept of trauma as both an individual and a collective phenomenon lies at the heart of this work. When individuals from divided communities participate in shared therapeutic spaces, trauma-informed interventions can promote not only internal regulation and personal healing but also the softening of social boundaries and the rehumanization of the “other” (Herman, 1997; Bar-Tal, 2000; Volkan, 2006). The impact of collective trauma on group identity, emotional climates, and openness to reconciliation has been extensively documented (Bar-Tal, 2000; Volkan, 2006; Hammack, 2006).

Within this context, peace psychology provides a complementary theoretical lens. Scholars such as Bar-Tal (2000) and Lederach (1998) emphasize that the transformation of protracted conflict requires addressing not only political structures but also the deep-seated cognitive, emotional, and narrative foundations that sustain division. Trauma-informed peacebuilding, a concept increasingly explored in psychological and peacebuilding literature (Alboszta, 2023), highlights emotional healing as a necessary precondition for rebuilding trust and fostering reconciliation.

Building on peace psychology, Lederach’s (1998) concept of “relational transformation” emphasizes that sustainable peace emerges from processes that rebuild human connections, empathy, and moral imagination—the capacity to envision the humanity of the other. In this sense, trauma-informed interventions such as EMDR can be understood not only as clinical techniques but also as relational practices that restore trust and promote mutual recognition. When professionals from both sides of a protracted conflict engage in shared learning about trauma and healing, they participate in a form of micro-level peacebuilding that aligns with Lederach’s call for grassroots, relationship-centered reconciliation.

Moreover, participating in joint professional training—particularly in fields dealing with sensitive subjects like trauma—can activate meaningful intergroup contact (Allport, 1954), especially under conditions of equality, common goals, and institutional support. Shared learning experiences may thus provide a platform for both professional development and incremental intergroup healing.

According to Allport (1954) intergroup contact theory, meaningful encounters between members of opposing groups can reduce prejudice and foster empathy when four key conditions are met: equal status among participants, pursuit of common goals, cooperative interaction, and institutional or societal support. The binational EMDR trainings organized by EMDR for Peace inherently fulfill these conditions—bringing together equally qualified professionals, united by a shared professional mission and supported by an institutional framework of trust and respect. Together, these conditions create an optimal environment for constructive contact, allowing professional collaboration to become a vehicle for humanization and attitude change.

Group-based EMDR training conducted across identity lines may therefore serve a dual function: enhancing clinical competence while simultaneously fostering interpersonal openness, empathy, and the reconstruction of relational models that contribute to both therapeutic and social healing.

Grounded in this integrative framework, this present study explores how these processes may manifest among students and professionals participating in a binational, bilingual EMDR training in the context of the Israeli-Palestinian conflict. Specifically, it examines participants’ self-reported professional self-efficacy, intergroup openness, and reflections on peacebuilding potential following the training experience. These dimensions are considered not only as markers of individual educational outcomes but also as potential indicators of broader contributions to social resilience in divided societies.

Taken together, these perspectives converge to suggest that trauma-informed professional training can serve as a bridge between personal transformation and intergroup reconciliation.

Through the integration of AIP theory with peace psychology and intergroup contact frameworks, this model offers a multidimensional lens for understanding the potential impact of EMDR training in divided contexts. Through the dual processes of trauma reprocessing and meaningful intergroup contact, participants may experience both professional growth and the reconstruction of empathy and trust, supporting the development of reconciliation readiness at individual and relational levels.

## Results

### Quantitative results

Descriptive results were examined to explore whether the shared EMDR training fostered perceived professional self-efficacy, intergroup openness, and reconciliation readiness—the three domains derived from the theoretical framework.

Descriptive and comparative statistics were calculated for the four quantitative items assessing these dimensions. All 24 participants provided valid responses across the four items.

**FIGURE 1:**
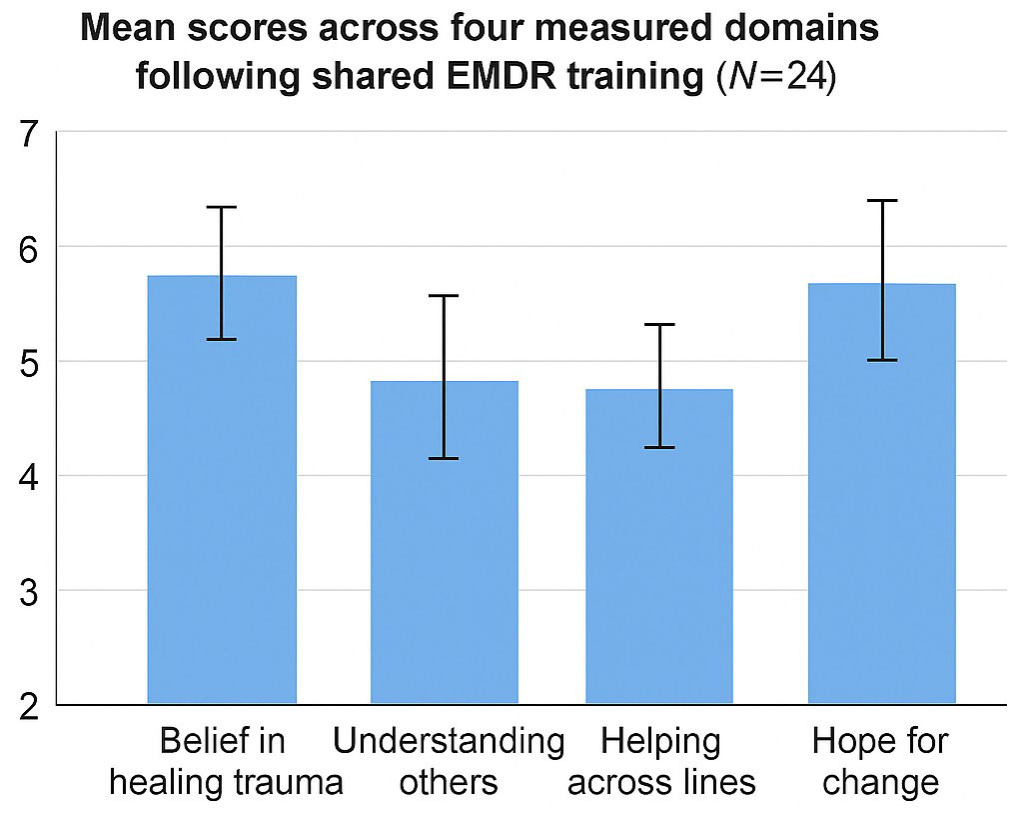
Mean scores across four measured domains following shared EMDR training (*N* = 24). Ratings were based on a 1–7 Likert scale (1 = *Strongly Disagree*, 7 = *Strongly Agree*). Values represent mean scores with error bars indicating ± 1 SD. Statistical comparisons between Jewish-Israeli and Arab-Palestinian participants were exploratory and conducted using independent-samples *t*-tests (equal variances not assumed).

Participants generally reported positive shifts following the training (see Table 2). Belief in the potential of trauma healing to contribute to reconciliation received a high mean rating (*M* = 5.67, *SD* = 1.20) on a 1–7 scale. Similarly, participants expressed hope that change is possible through shared therapeutic work (*M* = 5.58, *SD* = 1.41) and enhanced perceived capacity to assist others across group lines using EMDR (*M* = 5.25, *SD* = 1.51). The item regarding deepened understanding of individuals from different backgrounds yielded a somewhat lower mean score (*M* = 4.08, *SD* = 1.98), reflecting greater variability among participants on this dimension (see Figure 1).

**TABLE 2:** Summary of quantitative results and group comparisons (*N* = 24).

Measure	Mean (SD)	Range	Jewish-Israeli M (SD)	Arab-Palestinian M (SD)	*p*
Belief in trauma healing as a path to reconciliation	5.67 (1.20)	3–7	6.08 (1.08)	5.25 (1.22)	0.09
Deepened understanding of people from different backgrounds	4.08 (1.98)	1–7	3.08 (1.78)	5.08 (1.68)	0.01
Increased perceived capacity to help across group lines	5.25 (1.51)	1–7	4.75 (1.76)	5.75 (1.06)	0.11
Hope for change through shared therapeutic work	5.58 (1.41)	2–7	5.33 (1.78)	5.83 (0.94)	0.40

Ratings were based on a 1–7 Likert scale (1 = Strongly Disagree, 7 = Strongly Agree). *p-*values are based on independent-samples *t*-tests (equal variances not assumed).

Exploratory *t*-tests were conducted to compare Jewish-Israeli and Arab-Palestinian participants. As shown in Table 2, a significant difference was observed for intergroup understanding, with Arab-Palestinian participants reporting higher levels of understanding of people from different backgrounds (*p =* 0.01). Other group differences were not statistically significant, though meaningful trends were observed across domains.

These quantitative findings suggest that while participants generally experienced professional and interpersonal growth, variations in intergroup understanding highlight the complexity of transforming relational attitudes in short-term interventions. The comparative analysis further indicated that Arab-Palestinian participants reported significantly higher scores in intergroup understanding (*p =*0.01), suggesting that shared professional environments may differentially influence participants’ experiences depending on sociocultural background. Nonetheless, across all domains, both groups demonstrated positive and convergent trends, reflecting enhanced confidence, hope, and readiness for collaboration.

### Subgroup observations

Exploratory subgroup analyses compared Jewish-Israeli (*n* = 12) and Arab-Palestinian (*n* = 12) participants across the four measured domains. As reflected in Table 2, Arab-Palestinian participants reported significantly higher scores on intergroup understanding (*p* = 0.01), suggesting that shared learning contexts may foster greater interpersonal openness among participants from communities that are often underrepresented in professional training spaces.

Trends in other domains were not statistically significant but followed meaningful patterns. Jewish-Israeli participants tended to rate slightly higher belief in trauma healing as a path to reconciliation (*M* = 6.08 vs. 5.25), whereas Arab-Palestinian participants reported somewhat higher hope for change and capacity to help across group lines. These findings are consistent with qualitative reflections showing that, while all participants experienced growth, the dimensions most salient to each group reflected their distinct sociocultural vantage points within the shared society context.

### Qualitative findings

Qualitative data were analyzed using an inductive thematic approach (Braun and Clarke, 2006). Two researchers independently reviewed participants’ open-ended responses, coded recurring patterns, and collaboratively refined the final themes to ensure consistency and credibility.

Thematic analysis of participants’ open-ended responses revealed four main themes describing their professional and interpersonal experiences during the shared EMDR training. Table 3 presents the four main themes identified in the qualitative analysis.

**TABLE 3:** Summary of qualitative themes identified in participants’ reflections.

Theme	Example quotes
Interpersonal connection	“Learning together with different people.”
Experiential learning and supervision	“Practice sessions and supervision.”
Hope and skepticism about peacebuilding	“It was reassuring.”/“We didn’t talk much about this subject.”
Suggestions for improvement	“Meetings for open dialogue and discussion.”

Themes were identified through inductive thematic analysis. Example quotes are illustrative and not exhaustive.

1. Interpersonal connection and human encounter.

Many participants emphasized the interpersonal connection and sense of shared humanity that emerged throughout the training. They described the opportunity to meet and learn alongside colleagues from diverse backgrounds as deeply meaningful:

*“The opportunity to see different people, from different backgrounds, learn together.”*

2. Experiential learning and supervision.

Participants highlighted the importance of hands-on practice and supervision in consolidating both technical and relational learning:

*“The practice sessions and supervision.”*

*“Theory and practice.”*

3. Hope and skepticism about peacebuilding.

Reflections revealed a nuanced mix of hope and skepticism. While some participants expressed renewed optimism about the potential for change through shared therapeutic work, others noted that the training remained focused primarily on technical learning rather than explicit intergroup dialogue:

*“It was reassuring.”*

*“We didn’t talk much about this subject.”*

*“I didn’t see that as the purpose but I enjoyed the training.”*

4. Suggestions for improvement.

Several participants recommended including more structured opportunities for dialogue and interaction across group lines to deepen the interpersonal exchange:

*“Meetings for open dialogue and discussion on the issues.”*

*“Maybe more mixing.”*

Together, these reflections illustrate how participants integrated personal, professional, and relational dimensions of learning.

### Subgroup differences

Preliminary review of qualitative responses also suggested subtle distinctions between groups. Jewish-Israeli participants tended to emphasize technical and professional aspects of the training—practice sessions, supervision, and theoretical material—whereas Arab-Palestinian participants more often referred to empathy, interpersonal openness, and reflections on how shared therapeutic work could contribute to reconciliation. These patterns mirror the quantitative subgroup findings, reinforcing the idea that while all participants experienced growth, each group’s emphasis reflected its sociocultural position within the shared-society context.

### Integration of quantitative and qualitative insights

The convergence of findings from both strands highlights a consistent pattern: participants reported measurable increases in professional self-efficacy and hope, alongside qualitative reflections emphasizing connection, empathy, and the desire for deeper dialogue. While quantitative data revealed more moderate gains in intergroup understanding, the qualitative narratives suggest that emotional safety and shared learning laid the groundwork for relational openness.

Interpreted through the lens of the AIP model and peace psychology, these results indicate that joint EMDR training may initiate the early phases of “shared reprocessing”—a process through which professional collaboration contributes to mutual recognition and trust rebuilding across conflict lines.

## Discussion

This mixed-methods evaluation investigated the impact of trauma-informed EMDR training, delivered within a shared society framework, on participants’ professional self-efficacy and openness to intergroup engagement. Quantitative findings indicated that participants generally endorsed the view that trauma healing can promote reconciliation and expressed increased perceived capacity to work across group lines. Hope for positive change through collaborative therapeutic work was also strongly endorsed. However, gains in perceived understanding of individuals from different backgrounds were more variable, suggesting that while technical professional skills may be enhanced through shared training environments, deeper attitudinal shifts may require additional interventions focused on intergroup engagement.

Recent developments in trauma-informed peacebuilding emphasize the dual necessity of addressing emotional wounds and building practical capacities for reconciliation. Alboszta (2023) demonstrates how trauma-informed practices are being integrated into peacebuilding initiatives across diverse conflict-affected contexts, highlighting the importance of combining clinical competence development with efforts to foster social healing.

Together, the quantitative and qualitative strands converge to illustrate that trauma-informed professional training can operate simultaneously at the technical and relational levels. In line with the integrated theoretical framework, these results suggest that adaptive reprocessing and meaningful intergroup contact may function in parallel—enhancing both professional competence and the capacity for empathy and mutual recognition.

These integrative findings extend the theoretical understanding of trauma-informed training by demonstrating that shared professional learning, can simultaneously activate mechanisms of adaptive information processing and intergroup contact. This dual process offers an empirically grounded link between individual healing and collective reconciliation.

Thematic analysis of qualitative responses further illuminated participants’ experiences, highlighting the centrality of interpersonal connection, the value of experiential learning, and the nuanced interplay between hope and skepticism regarding intergroup peacebuilding. While many participants valued the opportunity to learn alongside individuals from different backgrounds, several expressed a desire for more structured opportunities to engage directly with identity and conflict-related issues. These observations align with prior research suggesting that meaningful intergroup contact requires not only shared activities but also intentional dialogue about identity, power, and historical narratives (Allport, 1954; Pettigrew and Tropp, 2006).

These findings also resonate with the Adaptive Information Processing (AIP) model (Shapiro, 2001), which posits that unresolved traumatic experiences can distort future perceptions and behaviors. From this perspective, trauma-informed training may contribute to reshaping relational schemas, not only on the individual level but also in terms of collective narratives (Bar-Tal, 2000; Volkan, 2006). Furthermore, narrative identity frameworks suggest that healing disrupted collective stories can facilitate shifts in intergroup perceptions and relational openness (Hammack, 2006). The concept of rehumanization (Volkan, 2006)—the restoration of empathy toward perceived outgroups—offers a useful lens for understanding participants’ experiences of emerging intergroup openness.

Moreover, this study reflects broader trends in trauma-informed education and peacebuilding literature (Miller and Rasmussen, 2020; Robinson and Kaptan, 2023), which increasingly recognize that addressing emotional legacies is a foundational step toward building sustainable peace. Professional training programs situated within divided societies thus carry a dual responsibility: fostering clinical competence while also creating the psychological conditions necessary for trust-building and conflict transformation.

Preliminary subgroup observations suggested potential differences in participants’ reflections based on ethnic background. Although the study was not designed for formal statistical comparisons, thematic patterns emerged. Jewish-Israeli participants tended to focus more on the professional and technical aspects of the training experience, often commenting on practice sessions, supervision, and theoretical learning, with relatively limited references to intergroup engagement or broader peacebuilding processes. In contrast, Arab-Palestinian participants more frequently emphasized experiences of empathy, the development of interpersonal openness, and reflections on the potential of shared therapeutic work to contribute to reconciliation. These preliminary trends highlight the importance of intentionally integrating opportunities for intergroup dialogue within professional training initiatives and warrant further investigation in larger, dedicated studies.

At the same time nevertheless, several limitations warrant caution. The sample was relatively small and self-selected, limiting generalizability. The absence of a control group precludes causal inference. One participant’s responses were excluded from the quantitative analysis due to inconsistency, and the lack of explicit guidance regarding the Likert scale direction may have introduced minor response bias. Furthermore, the study captured participants’ reflections immediately post-training, without assessing the durability of any reported changes over time. Finally, while the training provided shared technical experiences, opportunities for direct engagement with intergroup dynamics were limited, which may have constrained the depth of interpersonal transformation.

Future research should examine longitudinal outcomes, assess variations across different program structures (e.g., inclusion of structured intergroup dialogue sessions), and explore how trauma-informed education can contribute to both personal and societal healing in conflict-affected settings. Developing training models that intentionally integrate technical skill-building with opportunities for meaningful intergroup engagement may offer a promising path toward reconciliation and resilience in divided societies.

This study contributes to the emerging intersection of trauma-informed therapy, professional education, and peacebuilding. By examining how shared EMDR training experiences influence both individual professional development and intergroup openness, the research bridges clinical and sociopolitical domains. Beyond its descriptive contribution, the study offers a conceptual model for integrating trauma reprocessing with intergroup contact in divided societies, highlighting the potential of professional training as a structured, scalable form of grassroots peacebuilding.

Taken together, these insights underscore the transformative potential of trauma-informed professional education not only as a vehicle for clinical growth but also as a scalable framework for fostering empathy, cooperation, and peace in societies marked by enduring division.

## Conclusion

This study demonstrates the potential of trauma-informed EMDR training not only to enhance clinical competence but also to foster interpersonal openness within conflict-affected societies. Through a combination of quantitative and qualitative findings, the research suggests that shared professional training can serve as a preliminary platform for rehumanizing intergroup relationships and promoting reconciliation. However, the results also underscore the necessity of intentional facilitation of dialogue and reflection on identity and conflict issues to maximize the broader social healing impact. Future initiatives should integrate structured intergroup engagement alongside technical skill-building to support both individual resilience and collective transformation. Continued research is needed to explore how trauma-informed education can contribute to peacebuilding efforts, particularly in contexts characterized by entrenched societal divisions.

Beyond its immediate findings, this study proposes a conceptual bridge between trauma-informed clinical education and grassroots peacebuilding. By framing professional training as both a site of technical learning and relational transformation, it offers a replicable model for cultivating resilience and empathy across divided communities. Future initiatives can expand this approach through longitudinal evaluation and comparative cross-regional research to examine its broader peacebuilding impact.

## Data Availability

The raw data supporting the conclusions of this article will be made available by the authors, without undue reservation.
